# Microstructure of High-Performance Aluminum Alloy Surface Processed by the Single-Excitation Same-Frequency Longitudinal–Torsional Coupled Ultrasonic Vibration Milling

**DOI:** 10.3390/ma11101975

**Published:** 2018-10-13

**Authors:** Chongyang Zhao, Xiaobo Wang, Bo Zhao, Feng Jiao

**Affiliations:** School of Mechanical and Power Engineering, Henan Polytechnic University, Jiaozuo 454000, China; zhaocy@hpu.edu.cn (C.Z.); zhaob@hpu.edu.cn (B.Z.); jiaofeng@hpu.edu.cn (F.J.)

**Keywords:** ultrasonic milling, microstructure, roughness, wettability, frictional wear

## Abstract

The high performance of parts is determined by the microstructure of the machined surface to some extent. Different processing methods have been used to construct different microstructures on machined surfaces; the effective improvement of the serviceability of parts has been the focus of research in the field of precision and ultra-precision machining. In the presented work, a microscratch was formed on the machined surface in ultrasonic assisted machining, and the surface microstructure of high-performance aluminum alloy processed by single-excitation rotational longitudinal–torsional coupled ultrasonic vibration (LTCUV) milling was investigated. First, the motion paths model of the cutting edge in the LTCUV milling were established; then, the single-excitation LTCUV milling system has been set up, and the acoustic performance of the LTCUV system was examined. The surface microstructure of aluminum alloy was processed by different machining techniques, and the effect of processing parameters on the surface microstructure and performance were investigated by the orthogonal design of experiment (DOE). The surface roughness was found to be proportional to the ultrasonic cutting speed and feeding rate. The surface roughness was mainly controlled by the ultrasonic amplitude, and the optimal surface quality corresponded to the ultrasonic amplitude of 4 μm. The cutting speed contributes greatly to the surface roughness. The water contact angle of surfaces obtained by ultrasonic processing was larger than that of surfaces achieved by the conventional processing, while the surface water contact angle was negatively related to the ultrasonic amplitude. Once the rotation speed exceeded a critical level, the ultrasonic amplitude exerted a negligible effect on the surface water contact angle. The cutting speed contributes the most to the water contact angle. The friction coefficients of surfaces treated by ultrasonic processing were lower than those obtained by conventional processing at constant processing parameters, while the friction coefficient was minimized at the ultrasonic amplitude of 4 μm. In the case of grease lubrication friction, the surface wear decreased with the ultrasonic amplitude, indicating the improved wear resistance of the processed surfaces. Similarly, the ultrasonic amplitude has the highest contribution rate to friction and wear.

## 1. Introduction

Aluminum alloys are widely used in aerospace due to their low density, high cost-effectiveness, and excellent thermal/electrical conductivity [1,2]. However, the processing of aluminum alloys requires a high cutting force and heat dissipation, given their high viscosity and severe plastic deformations, and results in a degraded accuracy and surface quality of the processed surfaces [3,4]. In ultrasonic-assisted milling, high-frequency ultrasonic vibrations are applied to process tools or components after the amplitude amplification by the ultrasonic horn, while residual materials are readily removed by ultrasonic waves due to their high and concentrated energy and high impacts [5,6]. In contrast to conventional processing, the ultrasonic-assisted one is a high-frequency pulse vibration processing that leads to regular microscopic texture units at the component surface, which can effectively enhance the wettability and friction coefficient of the component [7,8].

Cutting force is an important parameter in machining. It can affect the surface quality of machined parts. Therefore, many mechanistic and empirical models have been used to model the cutting process due to their importance for industrial applications [9,10,11]. Przestacki et al. [12] studied the relationship between minimum uncut chip thickness and cutting force during machining. However, ultrasonic waves can change the chip thickness and thus change the cutting force [13]. Therefore, one of these technologies, which can be applied for the finishing of hard and brittle materials, is ultrasonic vibration cutting [14,15]. Ko et al. [16] found that it was helpful to improve the surface quality and stress, while appropriate feed per tooth was adopted in ultrasonic vibration milling. Ahmed et al. [17] developed a rotary ultrasonic system in the milling of alumina, and a lower cutting force and better surface quality were obtained.

The requirements for an excellent surface microstructure have led to extensive studies in the fields of process control and manufacturing process monitoring. Various processing methods have been utilized to obtain excellent surface microstructure, such as processing under cooling conditions [18], laser polishing [19,20], rapid solidification [21], and the optimization of the machining process [22,23]. Recently, the excellent microstructures of surfaces obtained by ultrasonic-assisted processing have attracted increasing attention. For example, the surface roughness of optical components with a hardness of HRC61 has been reduced by elliptical vibration cutting [24,25]. Chaises et al. [26] found that removing the residual tensile stresses and enhancing the compressive ones after ultrasonic shot peening improved the mechanical performance, corrosion resistance, and fatigue strength. Ko et al. [27] investigated the surface roughness by applying ultrasonic vibrations to the tool along the feeding direction. The results indicated that the quality of the ultrasonic vibration-processed surfaces was controlled by the cutting edge radius and the feeding rate per tooth. Babitsky et al. [28,29] performed the processing of aerospace materials such as Ni718 and C263 by ultrasonic-assisted and conventional turning methods. The results indicated that the surface quality of samples processed by the ultrasonic-assisted turning was improved by 25% compared to those produced by traditional turning. Some substructures were formed after ultrasonic surface rolling process treatment such as refined grains, dislocation walls, and deformation twins, which improved the modified surface’s hardness and resistance to shear deformation [30].

The above brief survey strongly suggests that considerable efforts were made regarding the implementation of ultrasonic vibrations and their impact on cutting force and surface roughness. However, the residual compressive stress on the material surface can be obtained by ultrasonic vibration-assisted machining, which can change the surface microstructure. Therefore, it is a meaningful work to explore the influence of ultrasonic processing methods and processing parameters on the surface microstructure. We propose a customized single-excitation rotational longitudinal–torsional coupled ultrasonic vibration (LTCUV) processing system and a testing platform on a DMG80 processing center. The aluminum alloys processed by LTCUV milling were subjected to the orthogonal test, and the treated surfaces were examined to clarify the LTCUV effect on their microstructure.

## 2. The System of LTCUV

### 2.1. Constitution of LTCUV System

**Figure 1:**
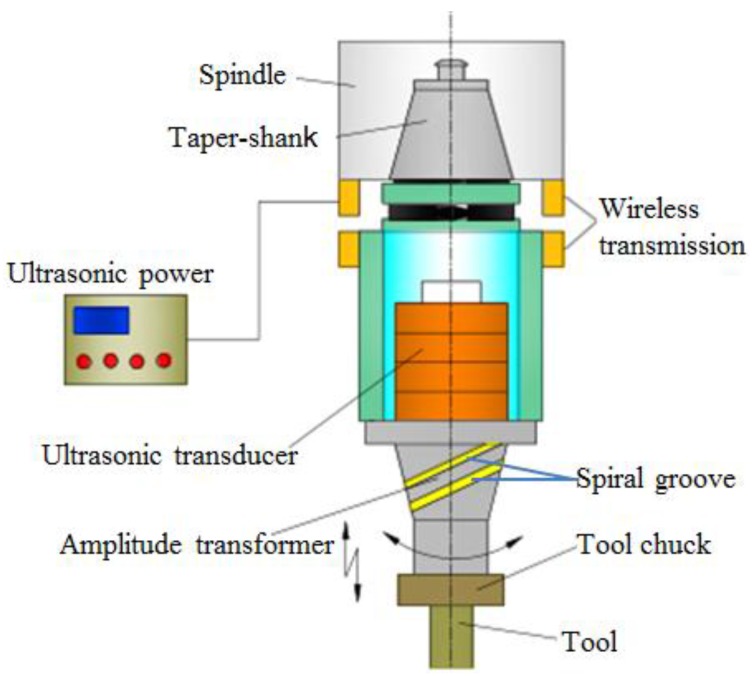
Longitudinal–torsional coupled ultrasonic vibration (LTCUV) milling system.

As shown in Figure 1, the LTCUV system is composed of ultrasonic power, electromagnetic induction wireless transmission, an ultrasonic transducer, an amplitude transformer, and a tool. The ultrasonic power supplies high-frequency electrical signals, while the electromagnetic induction launch plate (upper plate) and reception plate (lower plate) transmit signals to the sandwich piezoelectric transducer. The electrical signals are converted into mechanical vibration by the transducer. Then, the longitudinal vibration converts into longitudinal–torsional vibration by the spiral groove structure of the horn. Thus, the single-excitation LTCUV milling system is realized.

### 2.2. Motion Path Model of Ultrasonic Longitudinal–Torsional Milling Tool

**Figure 2:**
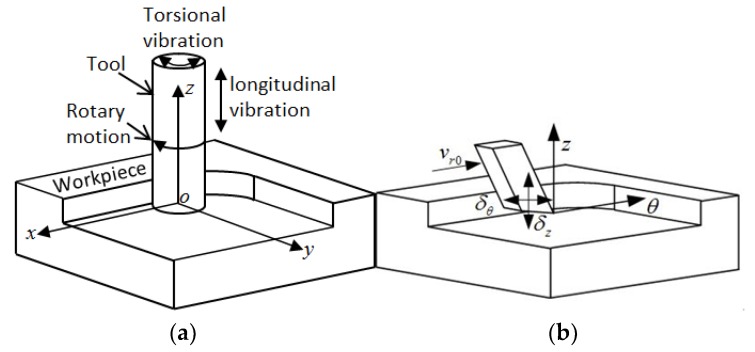
LTCUV milling mechanism model.((**a**) LTCUV milling model;(**b**)Cutting model of milling tip in axial and circumferential direction.).

Figure 2a shows the motion model of a cutting tool in LTCUV milling. During the processing, the milling cutter is exposed to gyration and longitudinal–torsional coupled high-frequency vibration simultaneously. As the gyration rate, longitudinal vibration speed, and torsional vibration speed of the end milling cutter were significantly larger than the linear feeding rate, the end milling cutter was expanded along its circumferential direction in the X–Y plane to elaborate a circumferential model for the end milling cutter, as shown in Figure 2b.

The axial and circumferential displacements of the milling cutter nose can be derived as follows:

(1) $$\left\{ \begin{array}{l} s=v_{r0}t+\delta_{\theta }\sin\omega t\\ z=-\delta_{z}\sin(\omega t+\alpha ) \end{array}\right.$$

The axial and circumferential speeds of the end milling cutter nose can be assessed as:

(2) $$\left\{ \begin{array}{l} v_{s}=v_{r0}+\delta_{\theta }\omega \cos\omega t\\ v_{z}=-\delta_{z}\omega \cos(\omega t+\alpha ) \end{array}\right.$$

In Equations (1) and (2), the term *ω* is the excitation frequency of the ultrasonic transducer, *α* is the phase difference between the longitudinal and torsional vibrations, *δ_Z_* and *δ_θ_* are the longitudinal and torsional vibration amplitudes, and *ν_r_*_0_ refers to the feeding rate of the milling cutter.

**Figure 3:**
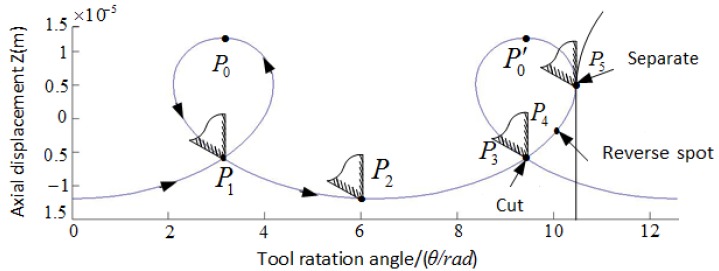
Trajectory characteristics of milling tip.

The motion path of the end milling cutter nose for the phase difference of 90° between the longitudinal and torsional vibrations is depicted in Figure 3. The milling cutter nose moved from P_0_ to P_0_′ via P_1_, P_2_, P_3_, P_4_, and P_5_ in one vibration period of LTCUV cutting. Each period consists of a cutting stage (P_0_–P_1_–P_2_), cutting stage (P_2_–P_3_–P_4_–P_5_), and separation stage (P_5_–P_0_′). In particular, the cutting tool is in contact with the sample in the period (P_1_–P_2_–P_3_–P_4_–P_5_), and is separated from it in periods (P_0_–P_1_) and (P_5_–P_0_′). Additionally, the friction between the cutting tool and the sample is reversed at P_4_.

According to the motion paths of the ultrasonic vibration milling tool, surfaces subjected to ultrasonic processing exhibited regular and compact microstructures, which may affect such surface characteristics as roughness, wettability, and frictional performance.

## 3. Methods and Results

### 3.1. Methods

The single-excitation LTCUV milling was applied to 7075 Al alloys using the triaxial vertical processing platform. The self-designed BT40 ultrasonic standard handle was excited using a single table, which was a self-improving ultrasonic source to achieve the single-excitation same-frequency LTCUV. Herein, the resonance frequency was 35,623 Hz, the longitudinal torsion ratio was 0.25, and the source power was 250 W. For a specific horn, as the longitudinal/torsional amplitude is constant, the overall amplitude was denoted as the longitudinal amplitude A.

**Figure 4:**
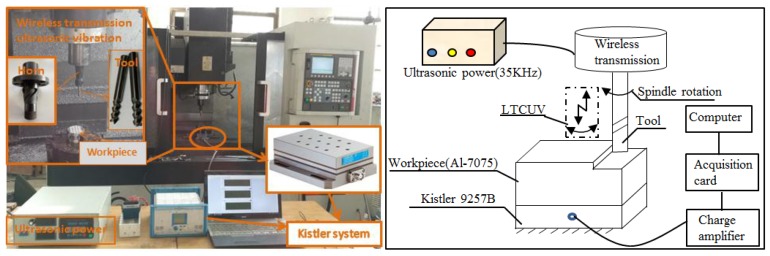
The experimental devices.

Table 1 summarizes the performance parameters of the 7075 Al alloys that were used in this study. Table 2 shows the geometrical parameters of the three-blade, flat head milling cutter for Al alloys. Figure 4 shows the experimental set-up. 7075 Al alloys and Kistler9257b are fixed on the worktable. The milling generates longitudinal and torsional vibrations by the LTCUV system. In the milling process, the milling force signal is examined by Kistler9257b. Then, the signal transmits from the charge amplifier to the acquisition card, and ultimately, the computer displays the milling force. Each experiment is repeated three times. The parameters of the dynamometer Kistler9257b are shown in Table 3.

Using suitable parameters and experiment methods could reduce cost and times. However, the frequencies of the LTCUV system and milling cutter have been determined through choosing the cutting speed, feeding rate per tooth, ultrasonic amplitude as influencing factors; the three-factor, five-level L25 orthogonal experiment was designed by the method of orthogonal design, whose parameters are summarized in Table 4. According to the experimental parameters, the 7075 aluminum alloy was milled by the LTCUV system, and the roughness, water contact angle, and friction coefficient were measured respectively in order to study the influence of the machining parameters on the cutting force, roughness, water contact angle, and the friction coefficient. The contribution rate of each machining parameter was obtained by measured values. It provided the basis for the selection of parameters in engineering application.

### 3.2. Impact of Processing Parameters on the Cutting Force and Surface Roughness

The processed components were sonicated to remove the residual oil. Firstly, the roughness of the milling surface Sa was measured using a non-contact three-dimensional (3D) white interference surface profiler (Taylor Surf CCI6000, Taylor Hobson Ltd., Leicester, United Kingdom). Herein, the average of five measurements was used as the final roughness value. Where, the sampling parameter is 5000 mm/s, the sampling area is 0.9 mm × 0.8 mm, and the sampling interval is 50 mm. The microstructure was examined using a VHX-1000 hyperfocal 3D microscope (KEYENCE Ltd., Itasca, IL, USA), which made it possible to investigate the relationship between roughness and microstructure.

**Figure 5:**
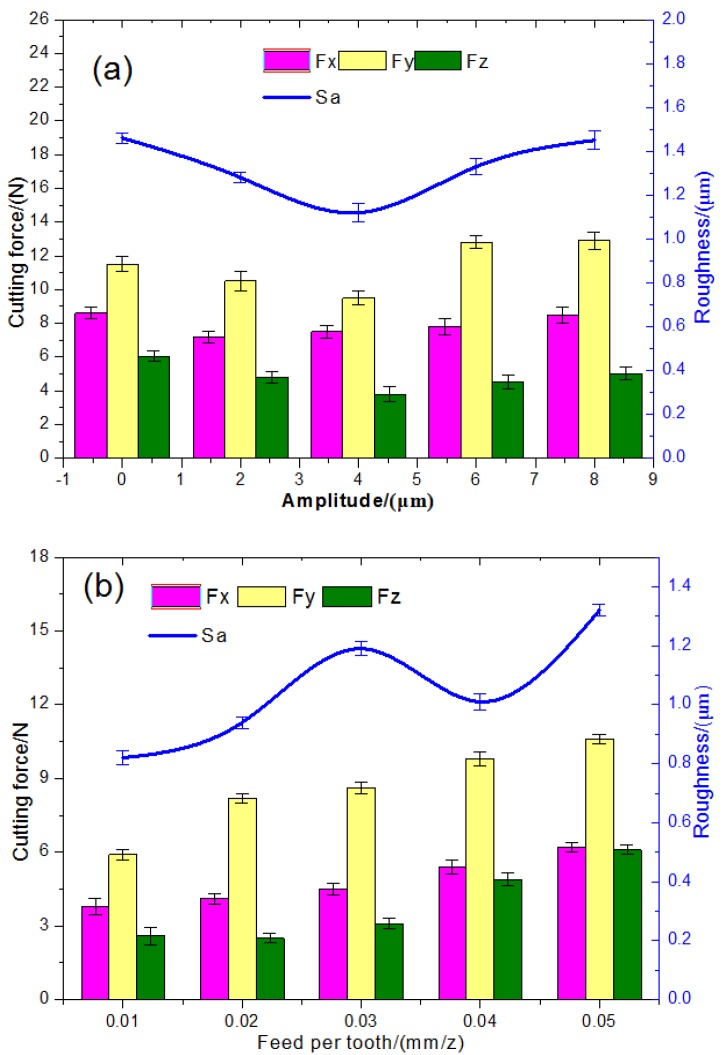

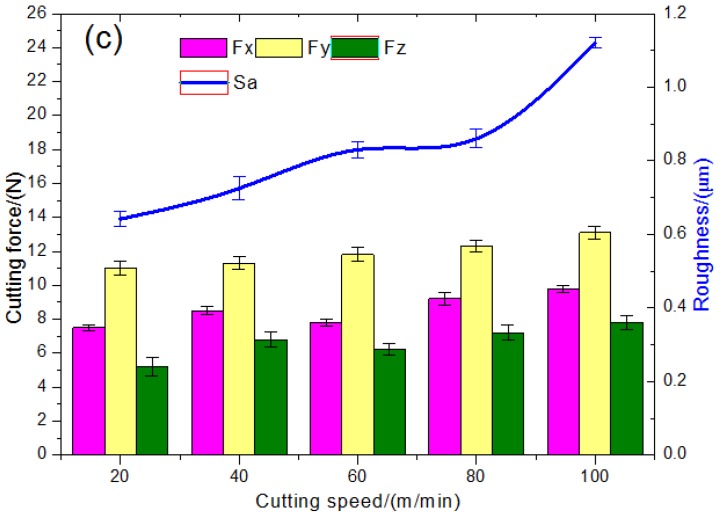
Parameters’ influence on cutting force and surface roughness ((**a**) v = 40 m/min, fz = 4 μm/z; (**b**) v = 40 m/min, A = 4 μm; (**c**) fz = 4 μm/z, A = 4 μm).

As shown in Figure 5a, the ultrasonic amplitude has a negligible effect on the milling force and a significant impact on the surface roughness. As the ultrasonic amplitude increased, the surface roughness dropped and then increased, with the maximal surface quality being attained at the ultrasonic amplitude of 4 μm. This can be attributed to continuous cutting being converted to high-frequency vibration-interrupted cutting due to the application of ultrasonic vibrations. As the impact of vibration cutting increased, the surface roughness was reduced. Nevertheless, the transient impacts increased drastically as the vibration amplitude was further increased, resulting in the degraded surface quality.

As seen in Figure 5b, the cutting force and surface roughness were proportional to the feeding rate per tooth. As the feeding rate per tooth increased, the resistance between the milling tool and the component increased, resulting in an increased cutting degree and average friction, which, in turn, leads to increased cutting forces. As a result, the microstructure varied as the feeding rate per tooth increased and the uniform tool marks were coarsened, resulting in increased roughness.

As seen in Figure 5c, the cutting force and roughness increased gradually with the cutting speed. At low-rotation speeds, the impact of a regular axial ultrasonic pulse per unit of time acts as the dominant factor for surface roughness. At high rotation speeds, the system stability degraded, resulting in increased surface roughness.

**Figure 6:**
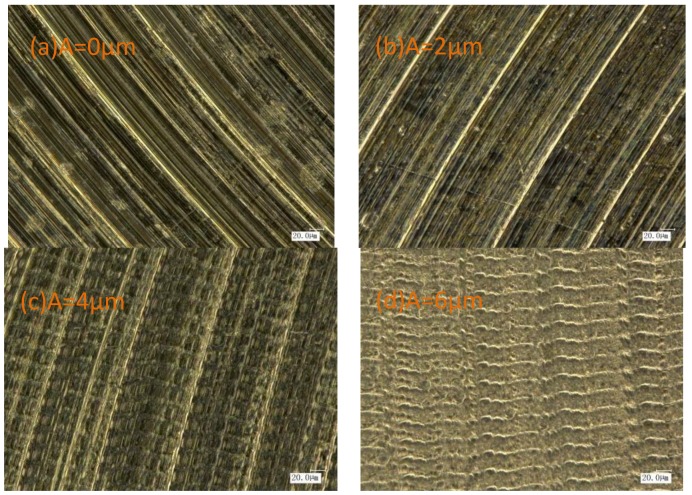

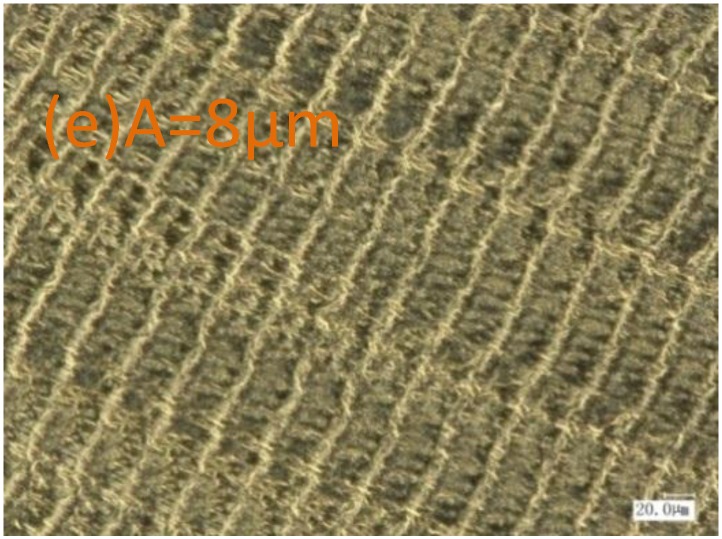
Microstructure under different amplitudes ((**a**) v = 40 m/min, fz = 4 μm/z, A = 0 μm; (**b**) v = 40 m/min, fz = 4 μm/z, A = 2 μm; (**c**) v = 40 m/min, fz = 4 μm/z, A = 4 μm; (**d**) v = 40 m/min, fz = 4 μm/z, A = 6 μm; (**e**) v = 40 m/min, fz = 4 μm/z, A = 8 μm;700 times).

Figure 6a shows the microstructure of samples subjected to the conventional milling. As observed, the motion path of the milling cutter was strongly manifested, with pits and grooves observed at the sample surface. As shown in Figure 6a,b, the motion path of the milling cutter at the ultrasonic amplitude of 2 μm was less pronounced, as compared to that observed in conventional processing. As seen in Figure 6c–e, uniform and compact networked textures were observed at the processed surface. These were exacerbated as the ultrasonic amplitude increased, indicating the increased edge heights and surface roughness.

### 3.3. Effects of Processing Parameters on Surface Wettability

#### 3.3.1. Effects of Processing Parameters on Water Contact Angle

The water contact angles of Al alloy surfaces processed by LTCUV milling were measured using the optical contact angle detector (OCA25) (DataPhysics Instruments GmbH, Filderstadt, Germany) to investigate the effects of microstructure on the surface wettability.

The surface water contact angle was measured using the distilled water droplet suspension method, wherein each droplet had a volume of 6 μL. Each test was repeated three times, and the average was regarded as the final value. Before being tested, samples were sonicated in acetone and dried to remove the surface impurities.

**Figure 7:**
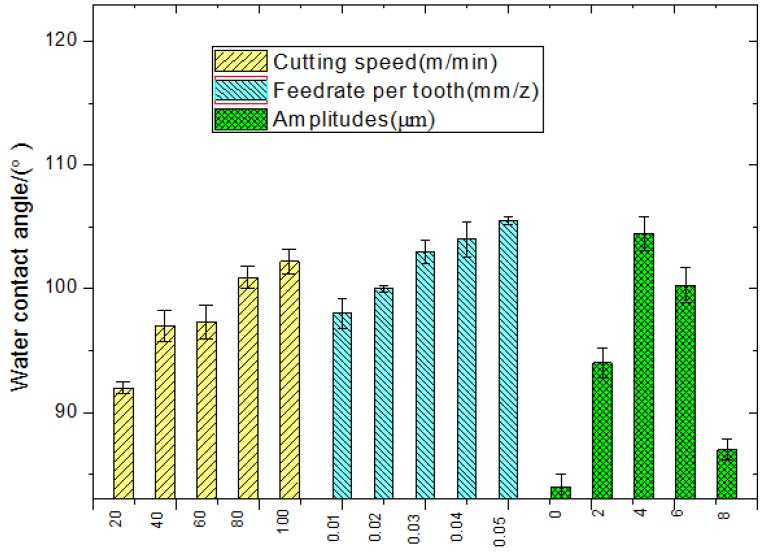
Parameters’ influence on water contact angle.

As shown in Figure 7, the cutting speed and the feeding rate per tooth were proportional, although variations were not significant. Herein, the ultrasonic amplitude plays a crucial role in the contact angle variation: at A = 0, the water drops tended to spread over the surface, and the water contact angle was equal to 84°; at A = 2 μm, the water drops tended to shrink, and the water contact angle was 94°; at A = 4 μm, the water contact angle was maximized. After that, the contact angle decreased with the ultrasonic amplitude.

#### 3.3.2. Ultrasonic Amplitude Effect on the Water Contact Angle

**Figure 8:**
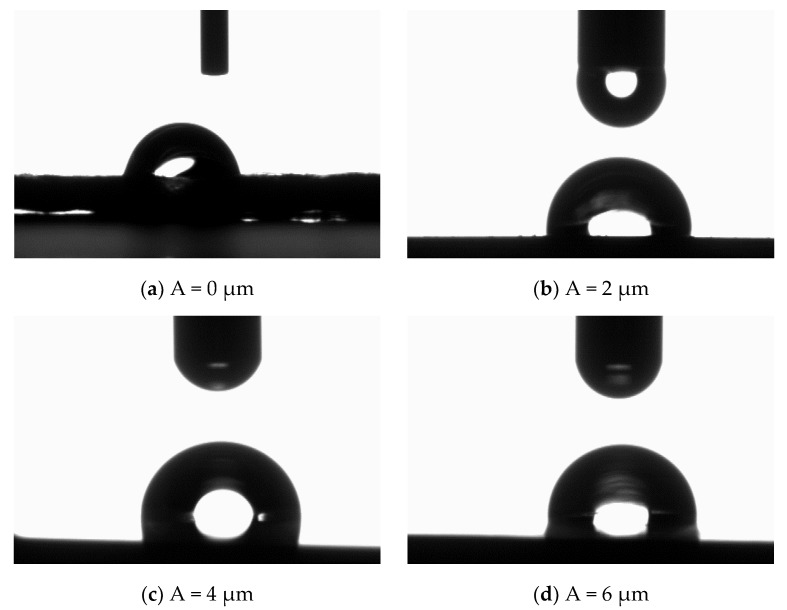
Water contact angle under different amplitudes ((**a**) v = 40 m/min, fz = 4 μm/z, A = 0 μm; (**b**) v = 40 m/min, fz = 4 μm/z, A = 2 μm; (**c**) v = 40 m/min, fz = 4 μm/z, A = 4 μm; (**d**) v = 40 m/min, fz = 4 μm/z, A = 6 μm).

Figure 8 shows the water contact angles of surfaces processed under different ultrasonic amplitudes. As observed, the surface water contact angle was affected by the ultrasonic-assisted milling: it increased with the ultrasonic amplitude and was maximized at the ultrasonic amplitude of 4 μm. This can be attributed to the increased surface roughness of Al alloys being induced by their periodically distributed compact microstructures, which, in turn, increased their surface wettability. In this way, hydrophobic metallic materials were prepared based on hydrophilic materials subjected to the ultrasonic-assisted milling without chemical modifications.

### 3.4. Wear Resistance of Surfaces Processed by Ultrasonic Longitudinal Torsion Milling

The frictional wear tests applied on Al alloys processed by LTCUV milling using a multi-functional frictional wear tester (WWM-1) (Blue wave laboratory, Jinan, China). Herein, the force was 10 N, the motion velocity was 3.8 mm/s, and the duration was 180 s. The quenching steel 45 with a hardness of HRC55 was employed as the contrast grinding material. The surface roughness Ra ≤ 0.02 μm was used for the polished arc contrast grinding bottom. The Ca-based lubrication grease was used as the grease lubrication in friction tests.

Before the tests, samples were sonicated in acetone for 20 min and then dried. Herein, the sample was coated with a lubrication grease, and the temperature was maintained at 20 °C. After the tests, samples were sonicated in acetone repeatedly until no residual abrasive dust or lubrication grease was observed. Eventually, samples were dried and tested by the hyperfocal detector for the measurement of frictional wear. 

#### 3.4.1. Effect of Processing Parameters on the Friction Coefficient

**Figure 9:**
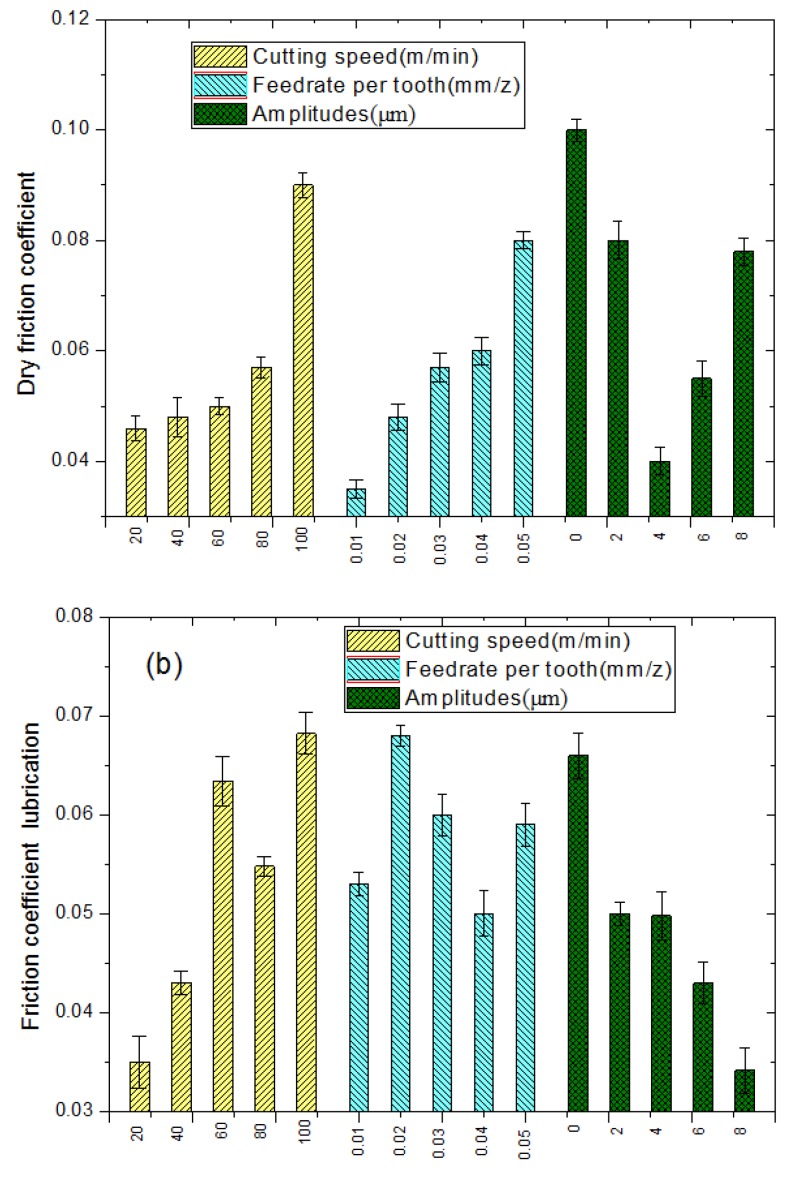
Parameters’ influence on friction coefficient ((**a**) Dry friction; (**b**) Lubrication friction).

As shown in Figure 9a, the friction coefficient increased with the cutting speed and the feeding rate. Smooth and compact surfaces could be obtained at low cutting speeds, low feeding rates, and appropriate ultrasonic amplitudes. In case of dry friction, the smooth and compact microstructure leads to an increased actual contact surface area of the friction pair, as well as the reduced suspension of the contact surface of the friction pair induced by the microscopic configuration waves on the surface. The latter, in turn, leads to lower friction and a reduced friction coefficient.

As seen in Figure 9b, large ultrasonic amplitudes lead to the improved lubrication characteristics of the processed surfaces at constant rotation speeds and feeding rates. This can be attributed to the high feeding rates enhancing the lubrication characteristics of the treated surfaces. At a constant feeding rate per tooth and ultrasonic amplitude, the friction curves at the three rotation speeds approached consistency during the friction progress. In general, the lubrication characteristics of the samples that were processed by LTCUV milling were superior to those of the samples treated by the conventional milling.

#### 3.4.2. Frictional Wear Microstructures of Samples Treated by Different Processing Methods

**Figure 10:**
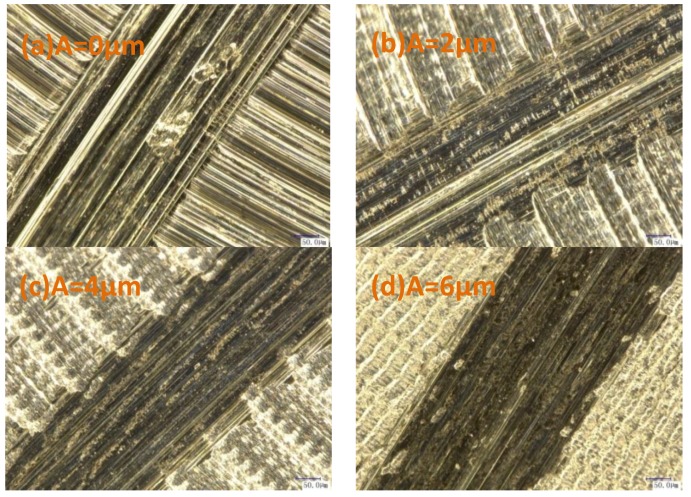
Wear microstructures ((**a**) v = 60 m/min, fz = 4 μm/z, A = 0 μm; (**b**) v = 60 m/min, fz = 4 μm/z, A = 2 μm; (**c**) v = 60 m/min, fz = 4 μm/z, A = 4 μm; (**d**) v = 60 m/min, fz = 4 μm/z, A = 6 μm; 500 times).

Figure 10 depicts the frictional wear microstructures of samples processed under different ultrasonic amplitudes. As observed, scratches in consistent directions with frictions were found on all of the surfaces, while those treated by conventional milling exhibited multiple severe scratches with a non-uniform distribution. In the samples treated by ultrasonic-assisted milling, surface scratches were reduced as the ultrasonic amplitude increased. The fine tool marks on the surface samples that were subjected to conventional milling implied their sliding against the contrast grinder, while the shear between the asperity peaks on the contrast grinder and sample surfaces generated the abrasive dust, which was pressed into the contact surface, resulting in surface grooves induced by shearing and cutting. The ultrasonic vibrations led to the uniform network; the contrast grinder was in contact with the network edges, and the asperity peaks at the contrast grinder surface were removed, thus reducing the number of direct scratches by asperity peaks at the contrast grinding surface. As the ultrasonic amplitude increased, the surface network was exacerbated, and the surface grooves were reduced.

**Figure 11:**
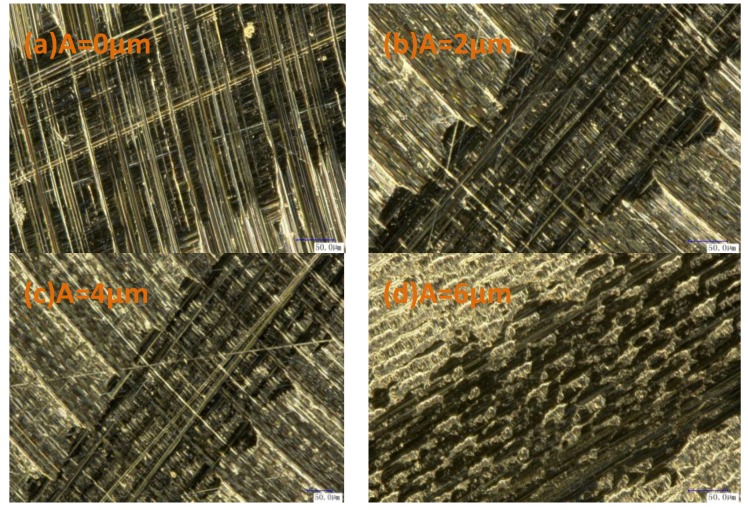
Friction and wear profile ((**a**) v = 60 m/min, fz = 4 μm/z, A = 0 μm; (**b**) v = 60 m/min, fz = 4 μm/z, A = 2 μm; (**c**) v = 60 m/min, fz = 4 μm/z, A = 4 μm; (**d**) v = 60 m/min, fz = 4 μm/z, A = 6 μm; 700 times).

As shown in Figure 11, at the ultrasonic amplitude of 6 μm, the network morphology of the milling surface was exacerbated, and the grease storage capacity of the pits at the surface increased. During sliding, friction pairs were compressed against each other, and pits were filled with sufficient lubrication grease. On the one hand, a thick grease film was developed at the friction surface, thus relieving the shearing between the peaks on the contact surface and maintaining the original microstructure. On the other hand, the lubrication grease can remove the abrasive dust from the contact surfaces, thus reducing the grooves induced by the abrasive grains.

### 3.5. Contribution Degree of Parameters to Microstructure

**Figure 12:**
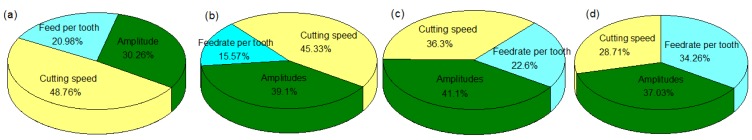
Contribution degree of parameters to microstructure ((**a**) to Sa; (**b**) to the water contact angle; (**c**) to the dry friction coefficient; (**d**) to the lubrication friction coefficient).

According to the DOE results, the contribution of different parameters to the surface microstructure was analyzed, as shown in Figure 12. The cutting speed has a great influence on the roughness and the water contact angle; its contribution rates are 48.76% and 45.33%, respectively. However, the ultrasonic amplitude is the great contribution to the dry friction coefficient and lubrication friction coefficient.

## 4. Conclusions

The motion paths of the cutting edge in LTCUV milling were studied, with the identification of regular and compact microstructures induced at the surfaces subjected to ultrasonic processing. Aluminum alloy samples processed by LTCUV milling were subjected to orthogonal tests, and the effect of the processing parameters on the surface characteristics was examined. The results obtained make it possible to draw the following conclusions.

(1) The surface roughness is proportional to the ultrasonic cutting speed and feeding rate, while the surface roughness is mainly controlled by the ultrasonic amplitude. The ultrasonic amplitude of 4 μm ensures an optimized surface quality.

(2) The water contact angle of the surfaces obtained by ultrasonic processing was larger than that of the surfaces achieved by the conventional processing, while the surface water contact angle was negatively related to the ultrasonic amplitude. Once the rotation speed exceeded a critical level, the ultrasonic amplitude exerted a negligible effect on the surface water contact angle.

(3) The friction coefficients of surfaces treated by ultrasonic processing were lower than those obtained by conventional processing at constant processing parameters, while the friction coefficient was minimized at the ultrasonic amplitude of 4 μm. In case of grease lubrication friction, the surface wear decreased with the ultrasonic amplitude, indicating the improved wear resistance of the processed surfaces.

## Figures and Tables

**Table 1:** 7075 aluminum alloy mechanical properties.

Tensile Strength (MPa)	Elongation Stress (MPa)	Elongation (%)
≥560	≥495	≥6

**Table 2:** Tool parameters.

Diameter (mm)	Helix Angle (°)	Cutter Teeth
8	55	3

**Table 3:** Kistler9257b parameters.

Rang Ability (KN)			Sensitivity (pc/N)		
Fx	Fy	Fz	Fx	Fy	Fz
±5	±5	±10	−7.929	−7.931	−3.712

**Table 4:** The test L25 (5^6) orthogonal table.

Number	Cutting Speed V (m/min)	Feed Per Tooth Fz (mm/z)	Amplitude A (μm)
1	20	0.01	0
2	40	0.02	2
3	60	0.03	4
4	80	0.04	6
5	100	0.05	8
